# Wearable fall risk assessment by discriminating recessive weak foot individual

**DOI:** 10.1186/s12984-025-01599-8

**Published:** 2025-03-20

**Authors:** Zhen Song, Jianlin Ou, Shibin Wu, Lin Shu, Qihan Fu, Xiangmin Xu

**Affiliations:** 1 School of Microelectronics, and School of EIE, South China University of Technology, Guangzhou, 510641 China; 2 The First Affiliated Hospital of Jinan University, Guangzhou, 510630 China; 3 Pazhou Lab, Guangzhou, 510330 China; 4 Department of Physics, Rensselear Polytechnic Institute, Troy, NY 12180 USA; 5 Institute of Modern Industrial Technology of SCUT in Zhongshan, Zhongshan, 528400 China; 6 Department of Biomedical Engineering, The Hong Kong Polytechnic University, Hong Kong, China

**Keywords:** Fall risk assessment, Wearable plantar pressure, Gait disorder, Weak foot, Machine learning

## Abstract

**Background:**

Sensor-based technologies have been widely used in fall risk assessment. To enhance the model's robustness and reliability, it is crucial to analyze and discuss the factors contributing to the misclassification of certain individuals, enabling purposeful and interpretable refinement.

**Methods:**

This study identified an abnormal gait pattern termed “Recessive weak foot (RWF),” characterized by a discontinuous high-risk gait on the weak foot side, observed through weak foot feature space. This condition negatively affected the training and performance of fall risk assessment models. To address this, we proposed a trainable threshold method to discriminate individuals with this pattern, thereby enhancing the model's generalization performance. We conducted feasibility and ablation studies on two self-established datasets and tested the compatibility on two published gait-related Parkinson’s disease (PD) datasets.

**Results:**

Guided by a customized index and the optimized adaptive thresholds, our method effectively screened out the RWF individuals. Specifically, after fine adaptation, the individual-specific models could achieve accuracies of 87.5% and 73.6% on an enhanced dataset. Compared to the baseline, the proposed two-stage model demonstrated improved performance, with an accuracy of 85.4% and sensitivity of 87.5%. In PD dataset, our method mitigated potential overfitting from low feature dimensions, increasing accuracy by 4.7%.

**Conclusions:**

Our results indicate the proposed method enhanced model generalization by allowing the model to account for individual differences in gait patterns and served as an effective tool for quality control, helping to reduce misdiagnosis. The identification of the RWF gait pattern prompted connections to related studies and theories, suggesting avenues for further research. Future investigations are needed to further explore the implications of this gait pattern and verify the method's compatibility.

## Introduction

Sensor-based technologies [1, 2] have shown the potential in screening fall risk factors and assess fall risk due to their objectivity, low cost, and ability to capture various human gait and posture signals [3]. Among existing studies (Supplementary Table. sI), accelerometers and inertial measurement units (IMUs) are the most common SRFT technology used in fall prediction (FP) [4, 5] and fall risk assessment [6–8]. Analyzing multi-domain acceleration data enables the detection of abnormal behaviors within a very short response time [4]. Howcroft et al. [9] found that a single accelerometer outperformed a pressure sensor in fall prediction tasks with the same sample length. Nevertheless, plantar pressure data remain valuable for addressing balance-related problems [10–14], locomotive stability [15–17], Parkinson’s disease (PD) [18–21] and other conditions [22, 23]. These technologies still hold great promise for long-term, low-obtrusive [3] fall risk monitoring, particularly when integrated into insoles or shoes [24–26]. The high-precision IMU, positioned near the center of mass [27], may interfere with the wearer’s daily activities, even when it is securely attached to the body with an elastic band for optimal comfort [6].

Although significant result has been achieved based on pressure pads with machine learning [28], the research on wearable shoe systems remains limited. In our previous work [25], the model based on wearable plantar pressure initially possessed the strong potential for long-term fall risk assessment with 87.5% accuracy. To further enhance the model's robustness and reliability across various scenarios and datasets, it is essential to analyze and discuss the factors contributing to the misclassification of certain individuals. This approach enables us to refine the model in a purposeful and interpretable manner, rather than simply focusing on engineering optimization. Moreover, there is a considerable variation in sample length among plantar-pressure-based works [18–23] (ranging from 0.5 to 5 min or 20 to 180 steps), as shown in the grey area in Table s1. Given the limited actual usage and the physical limitations of the elderly, it is essential to investigate how sample length impacts features and model performance.

To solve the problem, we chose a bottom-up, phenomenon-driven way to refine the model for fall risk assessment [25]. We proposed an adaptive threshold method and embedded it into a two-stage model. Several experiments were conducted on the proposed model’s effectiveness, robustness, interpretability, and compatibility. Compared to our previous work [25], the contributions can be summarized as follows:

- In addition to discriminating weak foot side as done in [25], we found variability in the performance of some individuals in weak foot features across distinct sample lengths.
- Based on the findings, we proposed an adaptive threshold method based on full-connection Euclidean distance of feature vectors to distinguish recessive weak foot (RWF) individuals, characterized by the discontinuous high-risk gait on the weak foot side.
- Two-stage model utilizing the adaptive threshold method outperformed the baseline model [25] by effectively accommodating individual differences in gait patterns. The threshold method served as an effective tool for quality control, helping to reduce misdiagnosis.

The paper is organized as follows. The data and features used in the subsequent section are introduced in Section II. The theoretical basis of the adaptive threshold method, structure, and implementation details of the two-stage model is described in Section III. Experiments and their results are detailed in Section IV. The results are discussed in Section V before the conclusion of Section VI.

## Data collection and processing

### A. Protocol and datasets

The details of the four datasets used in this study are presented in Table 1. In addition to the existing dataset I [29] for fall risk assessment, we established additional dataset II using the same protocol as dataset I to further evaluate the method’s robustness. In dataset II, a convenience sample of 32 older adults was recruited from the inpatients in the Rehabilitation Department of First Affiliated Hospital of Jinan University, with the age of 65 years or older, who can walk for over two minutes independently. The First Affiliated Hospital of Jinan University approved all the experimental procedures in this study (KY-2020–087) on Dec 24, 2020, and all the subjects read and signed the informed consent.

**Table 1:** Information of datasets

Subjects	Gender (M/F)	Age (years)	BMI (Kg/m^2^)
dataset i for fall ris﻿k assessment (Hu et al. [29])			
24 Low fall risk	10/14	72.3 ± 6.0	23.5 ± 2.9
24 High fall risk	15/9	75.9 ± 6.9	23.5 ± 2.8
Dataset II for fall risk assessment (This study)			
16 Low fall risk	9/7	71.8 ± 5.3	21.7 ± 3.3
16 High fall risk	12/4	75.4 ± 7.1	22.3 ± 4.1
Dataset III for PD (Yogev et al. [31])			
18 Controls	10/8	71.6 ± 6.7	25.9 ± 5.1
29 PD patients	20/9	71.1 ± 8.1	25.9 ± 3.7
Dataset IV for PD (Frenkel-Toledo et al. [32])			
29 Controls	18/11	57.9 ± 7.0	26.1 ± 3.7
35 PD patients	22/13	61.6 ± 8.9	25.2 ± 4.2

*PD* Parkinson’s disease

Before data collection, the Berg balance scale (BBS) test was performed on each participant. Participants were identified as high risk (HR) of fall if their BBS scores were less than 40 [30], and the rest of them were identified as low risk (LR) of fall. A prepared intelligent footwear system [24] with 16 pressure sensors distributed in different positions of the sensing insole was used to collect the plantar pressure of each foot. The participants were asked to walk for at least two minutes consecutively with their normal gait and speed in the 20-m-long corridor.

To assess proposed method’s scalability in other gait-related tasks, we conducted additional experiments on two plantar pressure-based Parkinson’s Disease (PD) datasets from PhysioNet [31, 32]. As shown in Table 1, gender, age, and BMI between the two groups was no significant difference (p > 0.05) as evaluated by the chi-square test and t-test.

### B. Data split and augmentation

**Fig. 1:**
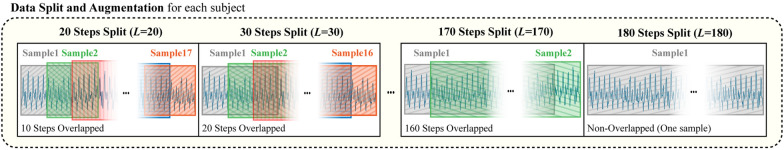
Data split and augmentation using step-level sliding window-based approach. *L* represents sample length

To reduce the impact of gait start-up while retaining valuable data, the first two steps of each subject were discarded [33]. The data was split and augmented by a step-level sliding window-based approach (Fig. 1). Firstly, to obtain the various sample lengths of different steps, data was split using different window lengths (20–180 steps) by identifying the zero value of foot plantar pressure. Specifically, sequential analysis and feature extraction are feasible in each window type due to time order and data continuity. Secondly, the window of length *L* slides forward by a fixed stride of *s* = 10 to create the next window until all sample points were included. As a result, the data from each subject can be divided into *N* samples using multiple windows The value of *N* can be calculated using Eq. (1):

1 $$N = (S - L)/s + 1$$

where S represents the total number of steps.

### C. Weak foot and its definition

In previous study [25], the concept of “weak foot”, referring one foot side that is functionally weak and has partially lost gait integrity, was introduced to enhance the predictive value of the extracted data variables while reducing the dependence of predictive models on extraction sides. Since the weaker foot side varies among individuals, features derived from distinct weaker sides are likely to carry more predictive value than those extracted from a fixed side [25], thereby accounting for individual differences.

We calculated sequence coordinates *(X*^*W*^*, Y*^*W*^*)* of Center-of-pressure (COP) across the entire window from the weak foot using Eq. (2), where *n* is the number of the pressure sensor, *F*_*i*_ and *(X*_*i*_*, Y*_*i*_*)* refer to the pressure value and relative coordinates of each sensor, and *Std*_*y*_ represents the standard deviation of COP in the anterior–posterior direction across.

2 $$(X^{W} ,Y^{W} ) = \left\{ {\begin{array}{*{20}c} {(\frac{{\sum\limits_{i = 0}^{n} {F_{i} X_{i}^{L} } }}{{\sum\limits_{i = 0}^{n} {F_{i} } }},\frac{{\sum\limits_{i = 0}^{n} {F_{i} Y_{i}^{L} } }}{{\sum\limits_{i = 0}^{n} {F_{i} } }}),Std_{y}^{L} < Std_{y}^{R} } \\ {(\frac{{\sum\limits_{i = 0}^{n} {F_{i} X_{i}^{R} } }}{{\sum\limits_{i = 0}^{n} {F_{i} } }},\frac{{\sum\limits_{i = 0}^{n} {F_{i} Y_{i}^{R} } }}{{\sum\limits_{i = 0}^{n} {F_{i} } }}),Std_{y}^{L} \ge Std_{y}^{R} } \\ \end{array} } \right.$$

### D. Feature sets

Forty-four COP features [25] based on weak foot extracted were:

- Weak and single foot features: Std_x_ (Standard deviation in medial–lateral COP), Mean_x_ (Mean in medial–lateral COP), Std_y_ (Standard deviation in anterior–posterior COP), Mean_y_ (Mean in anterior–posterior COP), MRD (Mean of resultant distance), SRD (Standard deviation of resultant distance), TOTEX (Total excursions), and CCA (Confidence circle area).
- Symmetry-based features: GAs (Gait asymmetry), SIM (Similarity), and JSD (JS-divergence).
- Temporal consistency-based features: GICs (Gait inconsistency), SSIM (Sequential similarity), and SJSD (Sequential JS-divergence).

## Methods

### E. Recessive weak foot individual

In this section, we determined the optimal sample length and investigated the factors contributing to misclassification of certain individuals through a pilot Leave-one-subject-out (LOSO) study. We identified and defined the recessive weak foot (RWF) individual, characterized by the discontinuous high-risk gait on the weak foot side. Drawing inspiration from the characteristics of RWF condition, we proposed an adaptive threshold method and a two-stage model.

**Fig. 2:**
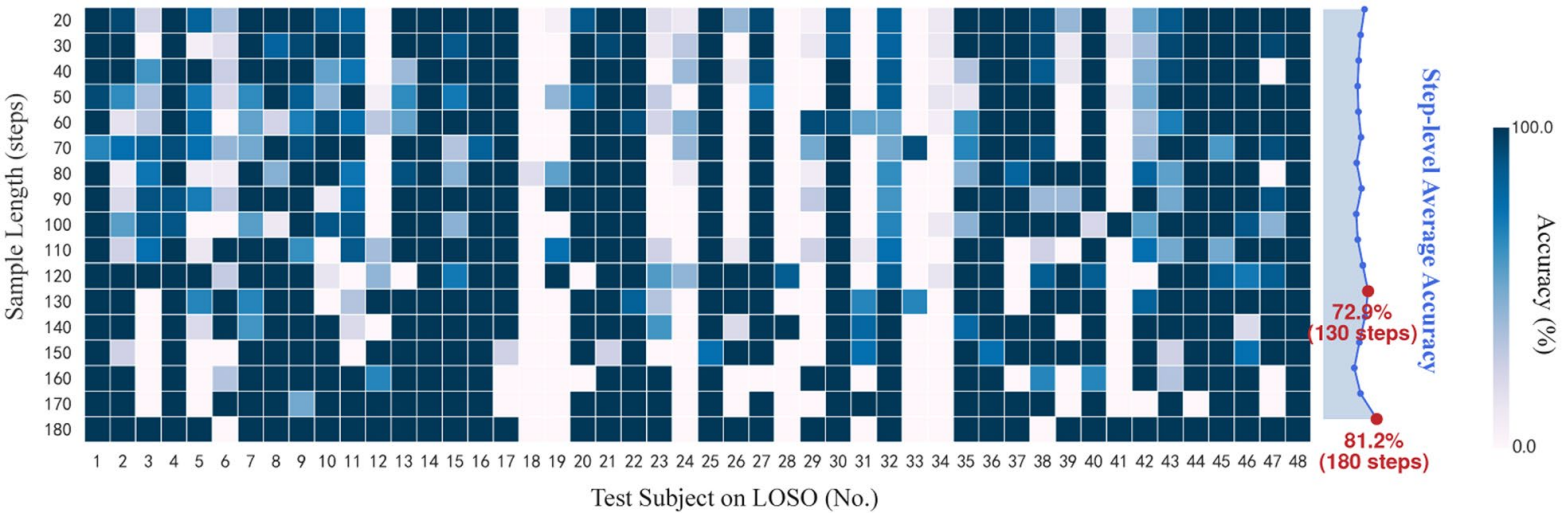
Overview results in the pilot study. The optimal sample lengths were determined to be 180 or 130 steps. *LOSO* leave-one-subject-out

*Theoretical basis* To investigate the impact of sample lengths and examine the causes of erroneous individual classification, we conducted a pilot LOSO study using Dataset I. The goal was to ensure that the fall risk assessment model utilizes as few gait samples as possible for accurate and timely testing of elderly individuals with limited data. Consequently, we employed a sliding window-based approach, resulting in repeated model selection and training with various LOSO training sets across different sample lengths (17 × 48 times), as illustrated in Fig. 2. Supplementary Fig. s2 shows that we conducted feature selection across 48 LOSO training sets and 17 different sample lengths, resulting in 816 optimal feature subsets. By observing the number of times different feature types were selected, we could understand the varying performance of features under different sample lengths. Finally, the optimal sample lengths were determined to be 180 or 130 steps (right side, Fig. 2). Due to the use of a sliding window-based approach, label inaccuracies arise from the fact that continuous samples from the same individual share the identical label. Consequently, as the sample length increases, the sample labels become more representative of the true risk levels, leading to the smallest label discrepancies and optimal model performance in the sample length of 180 steps. Two types of features presented opposite trends during the feature selection, achieving a balance near the second optimal lengths 130 steps (Supplementary Fig. s3). As the sample length increased, the temporal consistency-based feature obtained a broader range of calculations, making them more likely to be retained in the feature selection. Conversely, an unexpected phenomenon was observed: weak foot features tended to be filtered out more frequently at higher sample lengths.

**Fig. 3:**
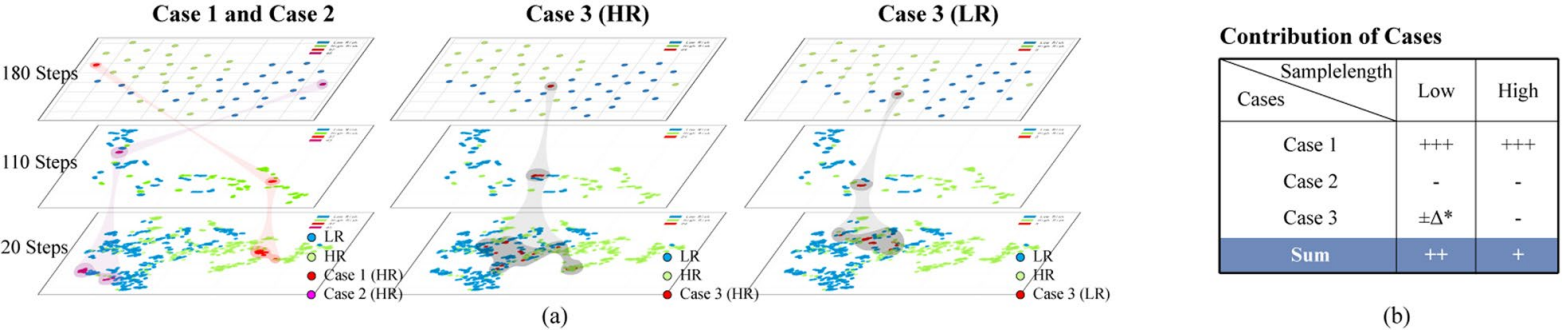
Contribution-based case analysis. **a** Three types of cases in weak foot feature space using t-SNE across different sample lengths (20, 110, and 180 steps). **b** Contribution of cases. “Contribution” refers to the influence of a sample on the model's generalization performance, which can be either positive or negative, depending on the sample's relative position within the feature space. ± Δ*: Small contribution. + : Positive contribution. −: Negative contribution. The number of signs represents the degree of contribution, which depends on the corresponding number of cases. *HR* High risk of fall, *LR* Low risk of fall

As seen in Fig. 3, a contribution-based case analysis was conducted to elucidate the unexpected phenomenon. Here, "contribution" refers to the influence of a sample on the model's generalization performance, which can be either positive or negative, depending on the sample's relative position within the feature space. Cases that contribute positively tend to cluster in adjacent areas, facilitating the construction of decision boundaries. In contrast, focusing on cases (with negative contributions) that are independent of the distribution of similar cases can lead to increased risk of overfitting. The degree of contribution depended on the number of cases. We defined and analyzed three cases of weak foot feature spaces under varying sample lengths to illustrate these dynamics. Based on previous study [25] and model dependency on feature numbers (Supplementary Fig. s1), we selected five weak foot features with the highest frequency of selection (Supplementary Fig. s2) from the pilot study to construct feature space via the t-SNE method [34]., Case 1 accounted for the largest proportion in feature space and made a significant positive contribution. In contrast, case 2 represented the smallest proportion and had a negative contribution, as they went deep into the area of another group. Importantly, the distributions of these two cases across different sample lengths were consistent and concentrated (red and purple areas), suggesting that they do not account for the observed variability in weak foot feature performance across different sample lengths.

The samples in Case 3 (grey areas) were dispersed across both HR (green) and LR areas (blue) at low sample lengths. Their contribution to the model's generalization performance depended on whether they clustered in areas with the same or opposite labels. Consequently, their effects often offset each other, resulting in a negligible overall contribution in low sample lengths. However, at higher sample lengths, these subject-level cases tended to occupy the border region between the two groups, increasing the likelihood of misclassification, particularly when treated as an independent unseen test set. Overall, weak foot features contribute more to model’ s robustness at lower sample length, as illustrated by the summed contributions of the three cases in Fig. 3(b), which explains the unexpected phenomenon of variability in weak foot feature performance.

*Recessive weak foot individual* Case 3 refers to individuals whose samples exhibit variability in the weak foot feature space, indicating inconsistencies in the gait of their weaker side. Those exhibiting this phenomenon were defined as “RWF individuals” due to the incomplete manifestation of high-risk gait characteristics on the weak foot side. In contrast, “Dominant weak foot (DWF) individuals” show relatively consistent weak foot performance, whether low-risk or high-risk.

### F. Adaptive threshold method

To mitigate the negative impact of RWF individuals to weak foot feature space, we aimed to propose a method for identifying RWF individuals. Based on the discontinuous characteristics of RWF individuals, we introduced a Distribution Difference Index (DDI) to quantify the degree of sample aggregation and dispersion at low sample length (*L*), serving as the reference for distinguishing RWF individuals. The DDI starts by calculating the high-dimensional Euclidean distance between each sample. Here, *v* represents the five-dimensional weak foot feature vectors mentioned in Section III. A. The full-connection distance *d* was calculated by averaging the distances between all feature vectors *v*. For RWF individuals, this distance *d* tends to be elevated at low *L*. Mathematically, the full-connection distance in a specific *L*, denoted as *d*_*L*_, is given by:

3 $$d_{L} = \frac{1}{{\sum {(N - 1)} }}\sum\nolimits_{i \ne j} {\left\| {v_{i} - v_{j} } \right\|}$$

where *N* represents the number of samples and can be deduced by the Eq. (1).

Due to individual differences, the gait variation among participants can differ significantly, leading to imprecision when comparing the full-connection distances between individuals. The samples at higher sample lengths were less affected by the RWF phenomenon. Hence, in the DDI calculation, the full-connection distance at high *L,* denoted as *d*_*h*_, is utilized as a baseline to eliminate individual differences at low *L*. Consequently, DDI is defined as:

4 $$DDI = d_{l} /d_{h}$$

where the value *d*_*l*_ was calculated at the smallest sample length to minimize excessive deviation between samples. In subsequent experiments, *d*_*l*_ was set to *d*_*20*_ as a compromise. The *d*_*h*_ was set according to the actual *L* of the dataset.

The discontinuous high-risk gait characteristics of RWF individuals result in higher DDI values. Driven inspiration from this principle, the DDI serves as a reference for distinguishing RWF individuals from DWF individuals through an adaptive threshold defined by:

5 $$T = DDI{}_{\min } + (DDI{}_{\max } - DDI_{\min } )\alpha$$

where DDI_max_ and DDI_min_ represent the maximum and minimum values of DDI in the training set, respectively, and *α* is a trainable parameter ranging from 0 to 1. Once the value of *α* is determined, individuals with a DDI greater than the threshold *T* are identified as RWF individuals. Importantly, DDI_max_ and DDI_*min*_ can be substituted with the upper and lower boundaries of outliers to ensure that *α* is adjusted within a normal range (e.g., DDI_max, min_ = μ ± 3σ, where μ and σ are the mean and standard deviation of DDI). The adaptive nature of this method is reflected in its trainable parameter *α,* enabling it to adapt to different tasks and datasets.

### G. Two-stage model and implementation details

**Fig. 4:**
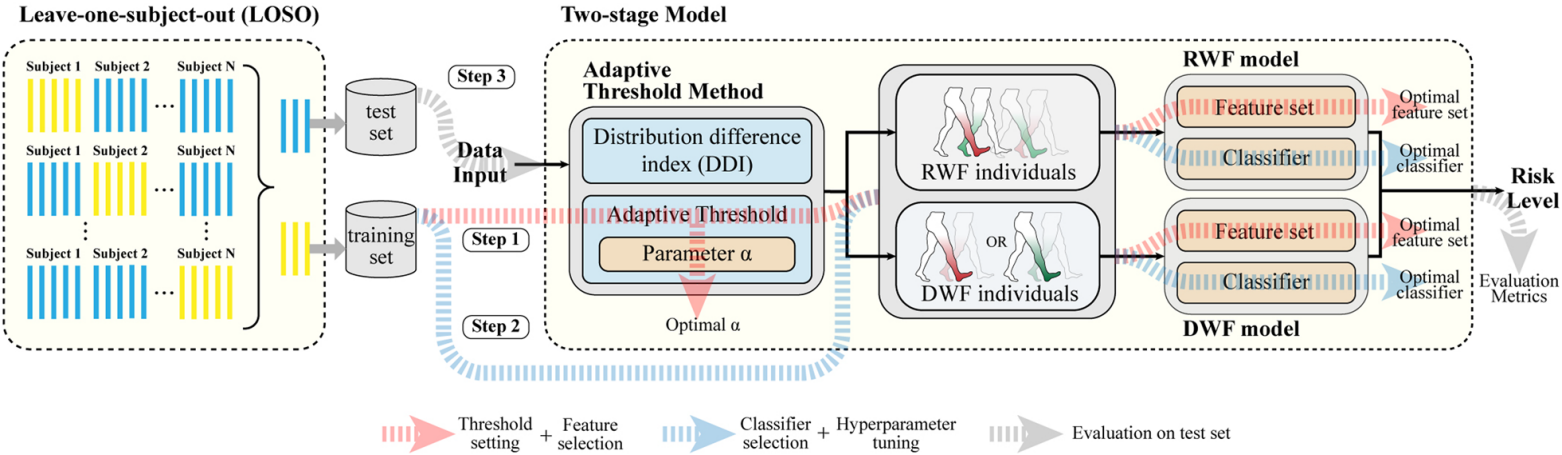
Architecture and implementation of the two-stage model. Step 1: Threshold setting and feature selection within the training set; Step 2: Classifier selection and hyperparameter tuning within the training set; Step 3: Model evaluation within the test set. *RWF* Recessive weak foot; DWF: Dominant weak foot

As shown in Fig. 4, we proposed a two-stage model that incorporated the adaptive threshold method. In the first stage, the adaptive threshold method distinguishes between two types of individuals. In the second stage, individual-specific models are trained for each type. The parameter *α* functions as a model parameter. When applying the adaptive threshold method to new data or new task, it is essential to retrain the two-stage model to ensure generalization.

As illustrated in Step 1 of Fig. 4, the optimal parameter α and feature sets for the two individual-specific models were initially selected based on the overall accuracy of the two-stage model in the training. Feature selection had three phases [25], namely pre-selecting based on Student’s t-test, multi-method selection (including five filter methods, four wrapper methods, and two embedded methods), and final selection. We fixed the stage-two classifiers with default hyperparameters in scikit-learn library [35] during step 1. Then the classifiers and parameters of the two models were selected and tuned in step 2. Seven candidate machine learning classifiers Logistic regression (LR), support vector machine (SVM), k-Nearest neighbor (KNN), decision tree (DT), random forest (RF), gradient boosting decision tree (GBDT), and AdaBoost were employed as candidates through scikit-learn implementation [35]. Two hyperparameters were tuned for each classifier, including misclassification cost and maximum iterations for LR, number of neighbors and leaf size for KNN, misclassification cost and gamma for SVM, maximum depth and minimum samples per leaf for DT and RF, and number of estimators and learning rate for GBDT and AdaBoost. The test set was unseen until it was used to evaluate the final two-stage model in step 3, preventing falsely inflated accuracy. Two evaluation metrics, accuracy and F1-score, can be calculated by:

6 $$Accuracy = (TP + TN)/(TP + TN + FP + FN)$$

7 $$F1 - score = 2TP/(2TP + FP + FN)$$

where TP, TN, FP, and FN represent True positive, True negative, False positive, and False negative, respectively.

## Results

### I. Method feasibility

To investigate whether the DDI-based threshold method can effectively identify the individuals unsuitable for the model, we divided the subjects into two groups based on the results of the pilot study (Fig. 2). Test subjects with an average accuracy exceeding 50% were classified as the high accuracy group (HA, n = 32), while those with an average accuracy below 50% were classified as the low accuracy group (LA, n = 16). It is important to note that this classification is distinct from the earlier references to high-risk and low-risk categories for falls. We performed Student’s t-test on DDI values to evaluate these two groups' differences. We employed the LOSO method on dataset I to evaluate the proposed two-stage model. A fall risk assessment model [25] with weak foot features was used as a baseline. We set sample length *L* to 180 steps, consistent with the baseline. To prevent the number of samples for RWF and DWF individuals from being too small, thereby risking overfitting in the second stage, we fixed the appropriate range of parameter α to 0.14–0.4 with an interval of 0.02.

**Fig. 5:**
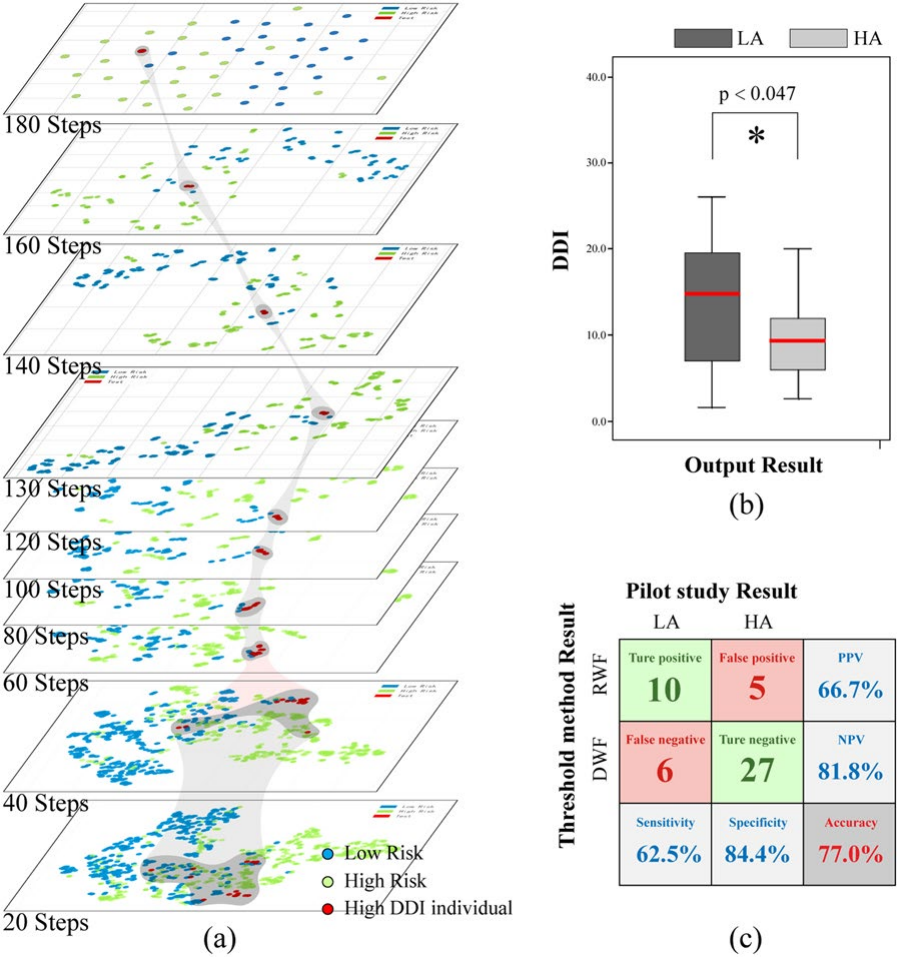
Performance of DDI and adaptive threshold method. **a** t-SNE visualization of the feature space for individual with high DDI. **b** DDI between LA and HA groups, with significant difference indicated by *. **c** Confusion matrix illustrating the effectiveness of the adaptive threshold method in distinguishing between LA and HA. *DDI* Distribution difference index, *HA* High accuracy group, *LA* Low accuracy group, *RWF* Recessive weak foot, *DWF* Dominant weak foot

**Fig. 6:**
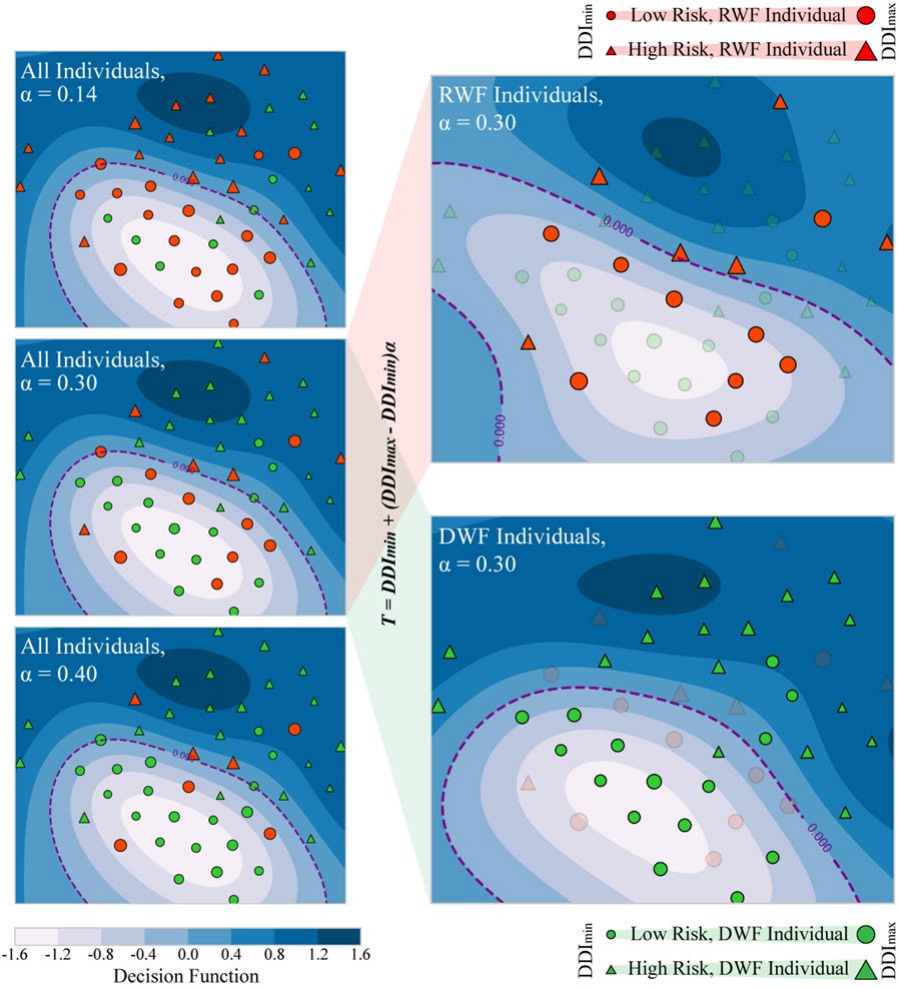
Analysis of adaptive threshold method. The size of the legends in the feature space represents the value of the DDI. The SVM classifiers with default hyperparameters along with the top five features selected in the pilot study were employed to generate feature space and decision function. Classifiers and features need to be reselected throughout the actual developing of the individual-specific model. RWF: Recessive weak foot; DWF: Dominant weak foot; DDI: Distribution Difference Index

*Adaptive threshold method* The feature spaces of high DDI individuals using the top 5 features in the pilot study (see Fig. s2) are shown in Fig. 5a. Due to the varying performance of weak foot features, there is noticeable dispersion of sample points among high DDI individuals at low sample lengths. As shown in Fig. 5b, the mean DDI of the LA group is significantly greater than that of the HA group (p < 0.05). Figure 6 shows the decision function, which denotes the signed distance between the sample and the hyperplane, thereby measuring the confidence score of the prediction. Individuals with high DDI values tended to cluster around the decision boundary, suggesting that they may belong to the LA group and could be misclassified by model. The adaptive threshold method can effectively identify RWF individuals by adjusting *α* values. As demonstrated in Fig. 5 (c), the accuracy of the DDI-based method with *α* = 0.3 reached 77%, indicating its efficacy in screening out the majority of RWF individuals from the LA group.

**Fig. 7:**
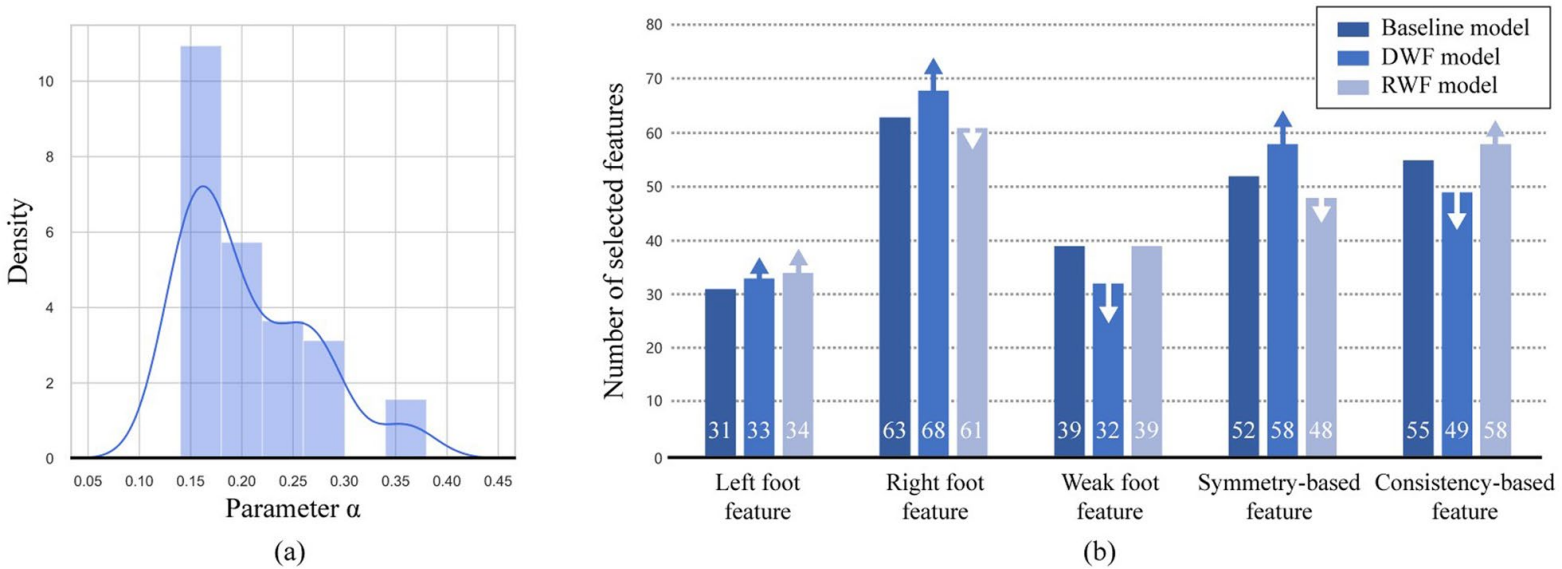
Results of the two-stage model development. **a** Density plot of selected parameter α among all LOSO iterations. **b** Adjustment of the optimal feature set from the baseline model. The arrow indicates the degree of increase or decrease compared to the one-stage baseline model. *RWF* Recessive weak foot, *DWF* Dominant weak foot

*Two-stage model* After the 48 iterations of LOSO training, nine test individuals were identified as RWF individuals across different LOSO iterations. During this process, a smaller parameter α resulted in a greater number of individuals being classified as RWF (left side, Fig. 6). Figure 7 (a) shows that the maximum value for the optimal parameter α reached only 0.36. while the lower limit of 0.14 was achieved in eight instances. Notably, when *α* was set to 0.14 in Fig. 6, over 60% of individuals were identified as RWF.

**Fig. 8:**
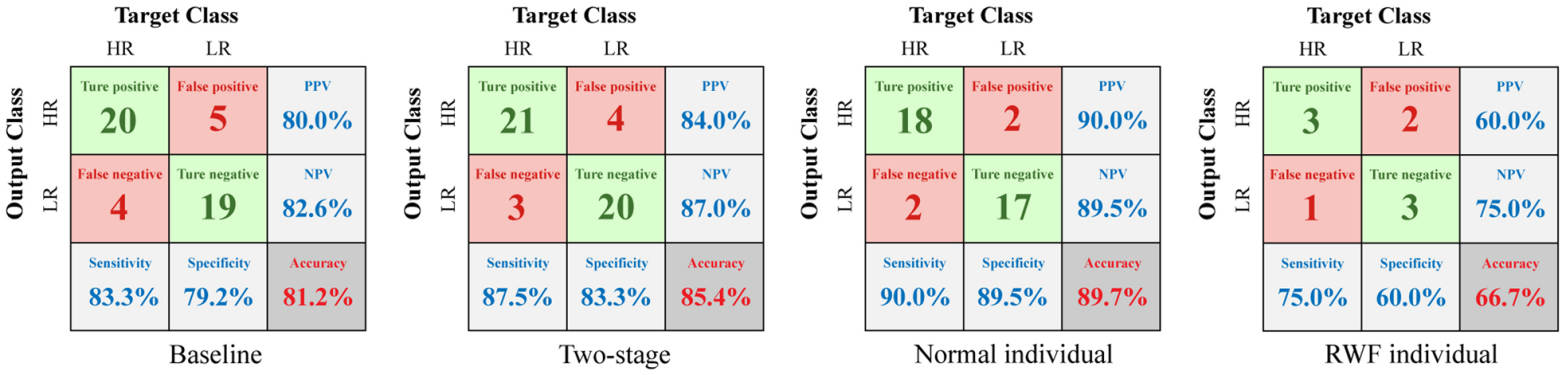
Confusion matrix on LOSO test set of dataset I. *RWF* Recessive weak foot, *DWF* Dominant weak foot

The fine adaptation of the baseline model for different individuals was mainly reflected in the training of individual-specific model and the adjustment of the optimal feature set. Taking the SVM classifier as an example (right side, Fig. 6), it established the optimal hyperplane based on the training samples from specific individuals. For the feature set adjustment shown in Fig. 7 (b), there were more significant changes in the number of selected feature types for DWF individuals, while RWF individuals exhibited relatively minor changes compared to the baseline. This limited variation for RWF individuals may be attributed to the training sample size. As shown in Fig. 8, the two-stage model achieved an accuracy of 85.4% with a sensitivity of 87.5%, compared to the 81.2% baseline accuracy. This improvement was largely attributed to the enhanced training set that excluded RWF individuals, allowing for better training of the DWF model, which reached an accuracy of 89.7%. Inadequate training of the RWF model might lead to overfitting and a significantly lower accuracy of 66.7%.

### J. Ablation studies

The introduction of the adaptive threshold method in the first stage, along with the individual-specific models in the second stage, complicates the understanding of the model's mechanisms and the sources of improvement. Since the pilot study and feasibility analysis were on dataset I, ablation studies were also performed on an additional dataset II using the LOSO method to investigate the method's behavior and evaluate the generalization performance without information leakage. To ameliorate the potential overfitting when developing the two-stage model on a small dataset (32 subjects), we adopted the second-best sample length of 130 steps for data augmentation. The sliding window-based approach enhanced the dataset sixfold (calculated by Eq. (1)), thereby increasing the resolution of evaluation metrics. This allows for more nuanced comparison across studies.

With data enhancement, the individual-specific models were fully trained even on the small dataset II, achieving accuracies of 82.1% and 85.5% on dataset I and accuracies of 87.5% and 79.2% on dataset II (top, Table 2). Despite label errors, the overall accuracy of 84.4% was slightly lower than the two-stage model under 180 steps. The F1-score, which emphasizes true positive cases and is more sensitive to recall, also highlights the improvements of the two-stage model over the one-stage model, with enhancements exceeding 10% on both datasets (top, Table 2), demonstrating the effectiveness and robustness of our adaptive threshold method. We conducted several ablation studies to interpret the improvements. The results of these two models served as baselines for the following comparison.

**Table 2:**
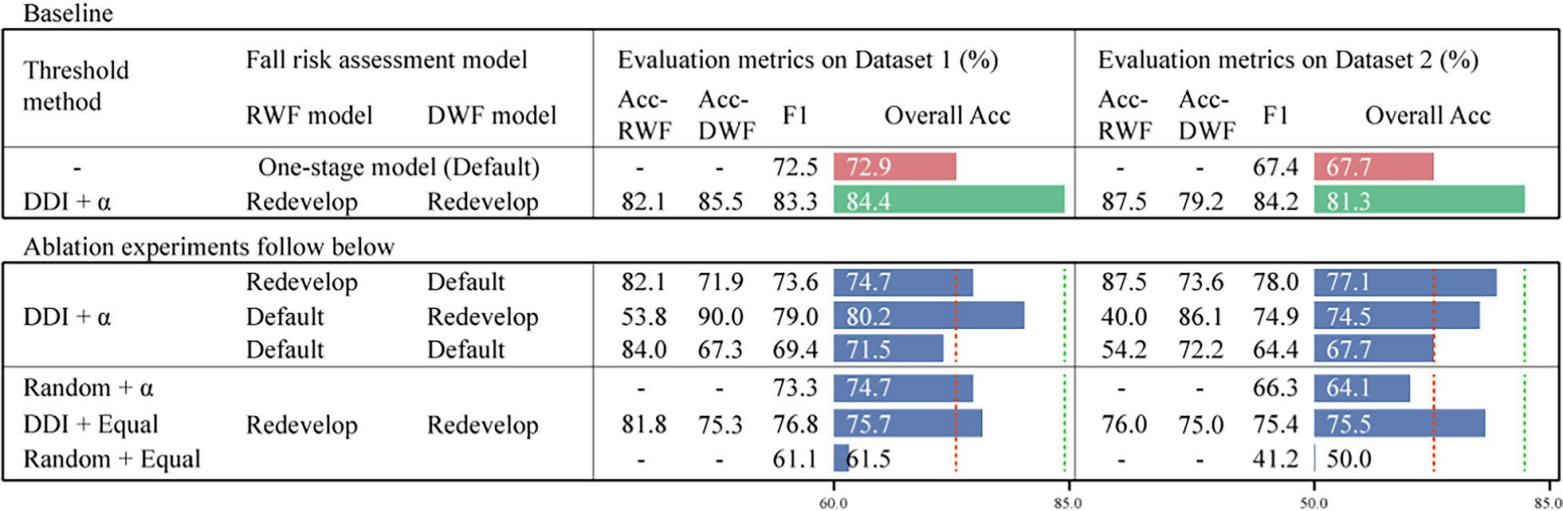
Results of ablation studies

The red and green dotted lines represent the baseline of the one-stage and two-stage models.: Random: We took the random number as the reference value, averaged over ten iterations. Equal: We preset the threshold to equally divide individuals. Redevelop: The individual-specific model was redeveloped with new features, classifiers, and parameters based on the training set. Default: The developed one-stage baseline model was used. *DDI* Distribution Difference Index; *RWF* Recessive weak foot; *DWF* Dominant weak foot; *Acc* Accuracy

First, we examined the impact of stage-two adaptation on individual-specific models. To do this, we stopped the developing process of the stage-two models and used the default features and classifiers selected in the one-stage baseline model instead. This deactivation led to varying degrees of decline in overall accuracy, dropping to 70.0% on both datasets when using the default model for both individual types. Given that this scenario is essentially akin to the one-stage model, the results were expected to be slightly lower than 72.9% on Dataset I and equal to 67.7% on Dataset II.

Next, we separately analyzed the roles of two key elements in the threshold method: the reference value and the threshold. We assessed the method's performance while eliminating one or both elements. For this purpose, we took the random number as the reference value, averaged over ten iterations, or preset the threshold to equally divide individuals. Masking the effect of the reference value (Random + *α*, Table 2) resulted in a 10% accuracy drop on dataset I compared to the two-stage baseline, with even poorer results on Dataset II than the one-stage baseline. In contrast, the model's performance improved when masking the effect of the adaptive threshold (DDI + Equal, Table 2). The worst outcomes occurred when both elements were masked, yielding 61.5% accuracy and 61.1% F1-score on Dataset I, and 50% accuracy and 41.2% F1-score on Dataset II. This highlights that the adaptive threshold, without either of the two key elements, is insufficient to effectively partition the appropriate and reasonable training set for the individual-specific model.

### K. Method compatibility

The adaptive threshold method cannot be used as a plug-and-play method, as DDI calculation is specifically limited to plantar pressure data. The RWF condition, representing an incomplete expression of gait integrity loss, may impact the results of other gait-related studies. To preliminarily explore the broader applicability of our method, we tested its compatibility with PD, a common balance-related [36] and fall-risk-related [37] disease. Alam et al. [19] developed various machine learning classifiers to distinguish PD patients from healthy controls, which we utilized as a baseline model for comparison. Their work did not divide the extra test set for evaluation, which may lead to inflated results [2]. To address this, in addition to dataset III for model selection, dataset IV was adopted as an external test set to evaluate the generalization performance. We conducted two reproduction procedures: Re 1, where we used ten selected features in the work [19]; Re 2, which involved using only the raw feature set along the feature selection method from their work [19], followed by reselection of optimal features.

Table 3 (top) shows the reproduction results of the baseline and our two-stage model on the training set. The optimal models from the training set were then tested on the external test set. As seen in Table 3 (bottom), the performances on the test set were generally poorer than those on the training set, indicating limited robustness of the model. However, by discriminating between different individual types and optimizing the training set in advance, we improved the model's robustness, increasing accuracy from 67.2% to approximately 71%.

**Table 3:** Results of method compatibility

Classifier	Alam 2017 [19]	Re 1	Re 2	Two-stage + Re 1		Two-stage + Re 2	
				RWF model	DWF model	RWF model	DWF model
Accuracy (%) on the training set (Dataset III)							
SVM	95.7	91.7	85.4	86.7	87.9	93.3	90.9
KNN	85.1	81.3	81.3	86.7	84.8	86.7	87.9
DT	87.2	85.4	83.3	86.7	81.8	93.3	81.8
RF	89.4	85.4	89.6	86.7	84.8	86.7	84.8
GBDT	–	85.4	85.4	66.7	81.8	66.7	84.8
AdaBoost	–	83.3	87.5	80.0	87.9	93.3	87.9
Accuracy (%) on the test set (Dataset IV)							
Selected model	–	67.2	67.2	83.3 (40)^a^	50.0 (24)^a^	72.0 (25)^a^	71.8 (39)^a^
				70.8		71.9	

We conducted two reproduction procedures: Re 1, where we used ten selected features in the work [19]; Re 2, which involved using only the raw feature set along the feature selection method from their work [19], followed by reselection of optimal features. ^a^The number of corresponding individual types. RWF, Recessive weak foot; DWF, Dominant weak foot

## Discussion

In this study, we identified a gait pattern “Recessive weak foot,” (RWF) characterized by a discontinuous high-risk gait on the weak foot side, observed through weak foot feature space. Individuals exhibiting this incomplete manifestation are defined as “RWF individual,” This condition negatively impacts the training and performance of common fall risk assessment model [25]. Hence, based on its pattern, we could propose a threshold method to identify those individuals to enhance the overall performance of the model.

Three existing interpretations, which are not mutually exclusive, may provide insights into implications of incomplete manifestation and help us understand potential reasons for model improvement: 1) Transition state: Liu et al. [38] considered samples located at the boundary between ill and normal areas as representing a transition state from illness to full recovery. This perspective suggests that relying solely on two risk levels in fall risk assessments is overly simplistic. The RWF condition provides a new avenue for exploration, particularly if the discontinuous gait also exists within this transition state. If this is indeed the case, it should be classified as a distinct category with significant characteristics, warranting further investigation. 2) Dual-task interference (DTI) [39]: Numerous studies [20, 40, 41] have shown that balanced or normal gait can occur in stroke patients during single-task experiments, with gait degradation typically manifesting only under dual-task conditions [39], such as motor-cognitive training. Normal gait relies on a precise coordination among various interacting neuronal systems [42]. When the automatic control provided by central pattern generators [43] is compromised by disease or injury, additional cognitive input can compensate for it, preventing abnormal gait from emerging under low cognitive load [41]. This theory suggests that some RWF states may involve cognitive compensation. Future study could investigate the performance of RWF individuals under dual-task conditions. Given the challenges posed by RWF individuals to the existing model, we should reconsider the incorporation of additional cognitive tasks into fall risk assessments during daily walking to enhance assessment accuracy. 3) Episodic gait disorders [42]: A notable characteristic of certain gait disturbances is their fluctuating or episodic nature, where specific provoking factors can differentiate these disturbances. The sudden and largely unpredictable changes in gait can manifest as loss of gait integrity across different episodes, closely consistent with the RWF condition. The RWF condition may serve as a significant indicator of episodic gait disorder in future studies.

In the feasibility study, we examined the effectiveness of DDI values, serving as a reference for identifying individuals unsuitable for classification based on weak foot features and validated the effectiveness of the DDI-based threshold method. As shown in Fig. 5, part of individuals unsuitable for the model could be effectively screened out by reference to DDI as the RWF phenomenon is generally associated with higher DDI values. The models tend to perform poorly with RWF individuals, while the low DDI in the LA group indicate that those individuals are often easily classified, typically exhibiting clear signs of their risk levels on the weak side. However, a few individuals with small DDI values remain in the LA group, primarily stemming from case 2 (Fig. 3). Due to their lack of apparent characteristics and absence of pathological basis for treating them as outliers, it is unproductive to attempt to improve the model using these cases. While individuals with high accuracy may be misidentified as RWF individuals (Fig. 5 (c)), there remains a significant likelihood of correctly classifying them in the second stage of classification. We only verified the feasibility of the adaptive threshold method since the above discussion shared the same dataset with the pilot study. Consequently, in the ablation experiment, we further tested the validity of the threshold method on a new dataset.

In the LOSO experiment, we observed that the adaptation of the baseline model for specific individuals improved the accuracy significantly. The high accuracy and sensitivity of our two-stage model is particularly important for early-stage screening of high fall risk, as it helps prevent missed diagnoses. This improvement was primarily attributed to the training of individual-specific models and the adjustment of the optimal feature set. During the training process, the DWF model was assigned fewer training samples, which was reflected in a tendency to select a lower threshold and utilize a larger training set for RWF model (Fig. 6 and Fig. 7a). This trend indicated that, after removing certain atypical individuals, the data characteristics of DWF individuals became easier for the model to learn. Conversely, the RWF model required more extensive training to capture the more variable data distribution of RWF individuals. The adaptation process from a general to a specific was not effectively realized in the RWF case. Inadequate training hindered the RWF model's ability to learn the unique characteristics of these individuals, resulting in feature selection that closely resembled the baseline model (Fig. 7b) and demonstrating poor predictive performance (Fig. 8). As a result, we implemented data augmentation in subsequent ablation studies. Additionally, in practical applications, a small number of RWF individuals could be regarded as outliers and manually diagnosed by medical personnel to enhance overall accuracy. Hence, the adaptive threshold could serve as an effective tool for quality control prior formal assessment, helping to reduce misdiagnosis, and optimize the use of medical resources.

In the ablation study, we demonstrated that each component of our model is essential for achieving improvement. The rules and theories derived from the pilot study are generalizable and not limited to Dataset I. When we halted the development, the accuracy of the undeveloped RWF model significantly dropped to 53.8% and 40.0% (Default + Redevelop, Table 2). This decline can be attributed to the model's lack of fine adaptation to individual characteristics, which is a crucial process in our two-stage model. In contrast, the accuracy of the undeveloped DWF model showed only a marginal deterioration of nearly 5%. This deterioration likely results from the inherent characteristics of DWF individuals being easier to learn and fit well even with a common model. The adaptive threshold method plays an important role in individual discrimination and quality control. Although a fixed threshold did not enhance the method's robustness, the DDI still provided valuable insights for distinguishing individuals who were more likely to be RWF. Ultimately, guided by the DDI and dynamic adjustments to the threshold, the adaptive threshold method effectively classifies individuals for different models. This tailored approach improves model generalization by allowing the model to account for individual differences in gait patterns, leading to more accurate predictions and reducing the risk of misclassification.

We preliminarily assessed the compatibility of our method using PD datasets. The biased raw feature set from [19] lacked consideration of spatial symmetry and temporal changes, easily resulting in overfitting. Additionally, ten of the thirteen features were selected as the final feature set, resulting in significant overlap among different candidate feature sets. Overfitting occurs when the model excessively learns the unique characteristics of specific individuals. By effectively discriminating between different individual types and optimizing the training set in advance, our two-stage model performed better. However, due to the low dimensionality of the raw feature set, the individual-specific model could not be finely tuned, which limited the extent of this improvement.

The adaptive threshold method cannot currently be implemented as a plug-and-play solution, as the DDI calculation is limited to plantar pressure data. It is essential to expand its applicability. Assuming that recessive RWF conditions may stem from impairments in the central pattern generator, gait disorders associated with neurological diseases [42] such as PD, stroke, Multiple Sclerosis and Spinal Cord Injury should be prioritized for further investigation. Subsequent studies could then explore other balance-related tasks, including scoliosis and knee osteoarthritis classification. Moreover, the method must be adaptable to a broader range of data types. To validate its transferability and scalability, we propose collecting various biological signals simultaneously, allowing us to explore and characterize the signal patterns associated with RWF individuals more comprehensively. Additionally, we intend to enhance the validation of our method's generalization by employing multi-center and cross-dataset analyses.

## Conclusion

This study defined individuals with the RWF gait pattern by analyzing weak foot feature space, proposing an adaptive threshold method to effectively discriminate RWF individuals. Embedded within a two-stage fall risk assessment model, this method was validated through feasibility and ablation studies on two self-established datasets and assessed for compatibility with two published gait-related PD datasets. Guided by DDI and optimized adaptive thresholds, our approach successfully screened RWF individuals, achieving accuracies of 87.5% and 73.6% on an enhanced dataset. Compared to the baseline, the two-stage model improved performance, demonstrating an accuracy of 85.4% and sensitivity of 87.5%. Additionally, in the PD dataset, our method reduced overfitting associated with low feature dimensions, increasing accuracy by 4.7%. These findings indicate that the proposed method enhances model generalization by accommodating individual gait differences, serving as a robust tool for quality control and reducing misdiagnosis. The identification of the RWF gait pattern has prompted connections to related studies and theories, highlighting the need for further research.

## Data Availability

Some results generated during this study are included in the Supplementary Materials.

## Abbreviations

IMU: Inertial measurement unit
FP: Fall prediction
PD: Parkinson’s disease
RWF: Recessive weak foot
DWF: Dominant weak foot
BBS: Berg balance scale
HR: High risk
LR: Low risk
LOSO: Leave-one-subject-out
DTI: Dual-task interference
DDI: Distribution difference index
LR: Logistic regression
SVM: Support vector machine
KNN: K-nearest neighbor
DT: Decision tree
RF: Random forest
GBDT: Gradient boosting decision tree
HA: High accuracy group
LA: Low accuracy group
